# Initial development and validation of a disease-specific resilience measure for inflammatory bowel disease: the RISE-IBD instrument

**DOI:** 10.1186/s12876-025-03971-3

**Published:** 2025-05-21

**Authors:** Michelle Mendiolaza, Camila Vicioso, Wamia Siddiqui, Karan Lingineni, Ksenia Gorbenko, Parul Agarwal, Laurie Keefer

**Affiliations:** 1 Institute for Translational Sciences, Icahn School of Medicine at Mount Sinai, 1 Gustave L. Levy Place, New York, NY 10029 USA; 2 Medical Education, Icahn School of Medicine at Mount Sinai, New York, NY USA; 3 Graduate School of Biomedical Sciences, Icahn School of Medicine at Mount Sinai, New York, NY USA; 4 Population Health Science and Policy, Icahn School of Medicine at Mount Sinai, New York, NY USA; 5 Institute for Healthcare Delivery Science, Mount Sinai Health System, New York, NY USA; 6 Division of Gastroenterology, Icahn School of Medicine at Mount Sinai, New York, NY USA

**Keywords:** Disease management, Inflammatory bowel disease, Psychometric validation, Resilience, Scale development

## Abstract

**Background:**

Inflammatory bowel diseases (IBDs) have a substantial effect on the well-being and quality of life of patients. While *resilience* may help alleviate psychological and physical burdens in individuals with IBD, there is no disease-specific measure that currently exists to accurately quantify this construct in the context of IBD. This study aimed to psychometrically validate the new Resilience Scale for IBD (RISE-IBD).

**Methods:**

A preliminary scale was developed including 17 items generated from a qualitative study that centered on patient focus group discussions, conversations with IBD healthcare providers, and evaluation of two validated resilience measures. In this cross-sectional study, the scale’s reliability, validity, and consistency were assessed in a sample of 91 patients with IBD.

**Results:**

The 17-item RISE-IBD was reduced to a 14-item measure. The revised RISE-IBD, comprising four resilience domains (Disease Acceptance, Self-Reliance, Flexibility, Persistence), demonstrated strong internal consistency (Cronbach’s α = 0.82) and excellent re-test reliability (*r* = 0.91). The scale exhibited significantly positive correlations with the Connor-Davidson Resilience Scale (*r* = 0.74) and Brief Resilience Scale (*r* = 0.59), as well as with a disease-specific quality of life scale, the IBD Questionnaire (τ = 0.33). It also showed significantly negative associations with psychological distress measured by the Brief Symptom Inventory (τ = -0.36) and IBD-related disability assessed by the IBD Disability Index (τ = -0.30).

**Conclusions:**

The RISE-IBD demonstrates its strong potential as a psychometrically reliable tool for assessing resilience in patients with IBD. By evaluating resilience across multiple domains, healthcare providers can gain a thorough understanding of patients’ coping mechanisms and tailor interventions accordingly. Future research should focus on further strengthening the scale’s psychometric properties by validating its use across diverse gastrointestinal patient populations and exploring the relationship between resilience and key IBD outcomes.

## Introduction

Inflammatory bowel diseases (IBDs), which encompass Crohn’s disease (CD) and ulcerative colitis (UC), are chronic immune-mediated conditions that adversely affect the gastrointestinal tract. IBD affect over 6 million individuals worldwide, with its prevalence rising in industrialized countries, including those in the United States (U.S.) and Europe [1]. The symptoms associated with IBD, including altered bowel movements, malnutrition, and extraintestinal manifestations, place a substantial emotional burden on patients, consequently affecting their health-related quality of life (HRQOL) [2].

Individuals with IBD frequently face daily coping challenges and difficulties with performing both physical and mental tasks, which can contribute to exacerbation of their psychological burden and perception of disease-related disability [3, 4]. Research underscores not only the heightened predisposition of individuals with IBD to anxiety and depression, but also emphasizes the likely role that psychological factors play in triggering clinical events or worsening physical symptoms in IBD patients [5].

Resilience, commonly defined as one’s ability to “bounce back” from adversity, has been recognized as a potential indicator of quality of life (QOL) in patients with chronic illnesses, including cancer, hypertension, and irritable bowel syndrome [6]. In a recent study, resilience was positively linked to mental and physical QOL in patients with IBD [7]. Additional research elucidates the notion that resilience may mediate the relationship between depression, anxiety, and disease activity in patients with IBD [8, 9]. These findings underscore the potential of resilience in improving health outcomes and managing disease-related challenges for individuals with IBD.

While therapies aimed at enhancing resilience for patients with chronic conditions have been associated with improved physical health and overall well-being, [10] only a few measures accurately quantify resilience in clinical settings [11–13]. Furthermore, despite the widespread use of the Connor–Davidson Resilience Scale (CD-RISC) [14] and the Brief Resilience Scale (BRS), [15] it is crucial to recognize that generic, patient-reported resilience measures may not fully capture the unique challenges faced by individuals living with IBD. These standard assessments may overlook the intricacies of managing IBD, such as dealing with unpredictable bowel movements, navigating dietary restrictions, coping with social stigma, and addressing specific mental health barriers, such as the anxiety of finding restroom access or the stress of adhering to complex treatment regimens.

Resilience in individuals with chronic conditions may be influenced by various factors unique to their situation, including their perception of the disease, adherence to medical and physical regimens, psychological functioning, and HRQOL [16, 17]. Given the highly heterogeneous nature of IBDs, there is a critical need for a patient-reported assessment tool that can accurately capture the nuanced experiences of individuals living with IBD to quantify this psychological construct accurately in this population.

The aim of this study was to provide preliminary evidence for the development and validation of a disease-specific resilience measure for IBD called the ***Resilience Scale for IBD (RISE-IBD).*** The tool is intended for use by clinicians and researchers to assess and monitor resilience in patients with IBD. By offering insights into the dynamic nature of resilience—accounting for its potential changes over time due to variations in disease status, medical and surgical treatments, or behavioral interventions—the measure can help identify patients who may require more focused interventions. It can also guide the development of tailored resilience-building management strategies with the long-term purpose of improving QOL for individuals with IBD.

## Materials and methods

### Study design

The study utilized a sequential-exploratory approach with mixed-methods consisting of two phases: (1) development of a draft of the RISE-IBD [18] and (2) psychometric validation of the RISE-IBD. The goal was to offer initial evidence for supporting the validity and reliability of a disease-specific resilience measurement tool for IBD.

### Development phase

A qualitative, patient-centered research study [18] was employed to explore the attributes of resilience as defined by individuals with IBD. Purposive sampling was used to recruit 15 adult patients who had been diagnosed with IBD for at least 3 months. Patients were required to achieve a rounded score of 3.00 on the BRS [15] indicating a moderate level of resilience. The research team developed a preliminary IBD resilience measure sequentially through 3 Focus Group Discussions (FGDs) with patients, and one member check discussion with 6 IBD healthcare professionals (comprising four physicians, one pharmacist, and one dietitian). All FGDs were conducted by focus groups or one-to-one interviews using an institutional HIPAA-compliant audiovisual platform and were audio-recorded.

During FGD 1, patients responded to nine open-ended questions aimed at uncovering the emotional, physical, and mental challenges associated with living with IBD, as well as the strategies patients used to navigate these hurdles. In FGD 2, patients were introduced to two established resilience measures, the CD-RISC [14] and the Resilience Scale (RS) [19], and were asked to assess the relevance of the themes depicted in these scales within the context of IBD. Following FGDs 1 and 2, the research team conducted thematic analysis to construct an initial version of the RISE-IBD. The thematic analysis methodology integrated aspects of the Grounded Theory [20] incorporating open coding to generate conceptual codes, followed by axial and selective coding. Axial coding facilitated connections among the open codes and selective coding was applied to interconnect these categories into themes that elucidated the construct of resilience. Independent and parallel coding of the transcripts was performed using the qualitative analysis software MAXQDA 2022 (VERBI Software, 2021). Following thematic analysis, a comprehensive codebook was devised containing 15 potential scale items.

In FGD 3, the participants and IBD professionals (i.e., member check) were invited to provide feedback on the drafted measure in two separate discussions. This thorough feedback approach was adopted to establish face and content validity of the selected items. Participants were asked to evaluate each item by reflecting on their pre-existing notions of resilience concerning IBD and asked to assess the clarity of the instructions provided for the drafted measure. Participants also provided feedback on Likert scaling, with the 5-level Likert rating method emerging as the preferred choice. Leveraging their professional insights, the member check allowed healthcare professionals to provide feedback on whether items were pertinent for inclusion, identify any potential redundancies, and offer suggestions for refinement of the drafted measure.

The feedback acquired from both groups led to considerable modifications of the scale, such as amendment to the wording of items, addition of a temporal component to the instructions, and the inclusion of two extra scale items. Consequently, the preliminary version of the RISE-IBD was comprised of 17 items organized into 3 theoretical domains: interpersonal fortitude, individual character strengths, and logistical strategies aimed at enhancing resilience.

### Validation phase

The psychometric attributes of the RISE-IBD, including the validity, reliability, and consistency of the scale, were assessed among adult patients treated at an urban tertiary care IBD center in the U.S.

#### Participants

Patients were recruited following their routine IBD clinical care visits and were contacted by phone from September 2023 to January 2024. The inclusion criteria encompassed patients who were 18 years of age or older, had an endoscopically confirmed diagnosis of UC or CD, had been living with IBD for at least 3 months, and were able to provide written informed consent in English. Participants provided self-reported demographic and clinical information regarding their condition. They were also asked to complete the initial RISE-IBD assessment along with five validated questionnaires as part of the validation process. Four weeks following the initial completion of the RISE-IBD, patients were invited to complete a re-test of the measure. A reminder email was sent after two weeks to remind participants to complete the re-test.

#### Data collection

All patients consented to participating in the study and completing all required questionnaires using an institutional HIPAA-compliant data-capturing platform. After completing each questionnaire, participants received detailed instructions via email to deter multiple responses from the same individual and ensure clarity regarding instructions. Participants were instructed to complete the questionnaires in a specific sequence, only once, and were encouraged to finish them within four weeks. To maintain patient anonymity, identifying information was removed from survey results. Subsequently, data was extracted from the data-capturing system into Microsoft Excel for analysis.

#### Experimental measure

The Resilience Scale for Inflammatory Bowel Disease (RISE-IBD).

The preliminary RISE-IBD is a disease-specific self-report measure of perceived resilience consisting of 17 proposed items. The 17 items are categorized into three domains: (1) interpersonal fortitude, (2) individual character strengths, and (3) logistical strategies. Instructions on the scale ask participants to reflect on the strategies they employ to manage their IBD when responding to the measure. Participants are expected to select the option that best aligns with their experiences over the past three months. Responses are given utilizing a five-point Likert scale ranging from 0 (“not true”) to 4 (“always true”). To mitigate social desirability bias, statements are positively and negatively worded, with reverse scoring employed for the latter. Scores on the preliminary measure range from 0 to 68, with higher scores indicating greater resilience.

#### Sociodemographic and clinical information

Participants were asked to self-report several demographic and illness-related variables using an online survey, which included multiple-choice and specific questions that allowed for multiple selections. The variables collected included age, gender, ethnicity, education level, marital status, IBD diagnosis, disease duration, disease activity status (flare versus remission), extraintestinal manifestations, comorbidities, medication regimen, frequency of physician appointments, and IBD-related surgical history.

### Validated measures

The Connor-Davidson Resilience Scale (CD-RISC) [14].

The CD-RISC is a validated 25-item self-report scale designed to evaluate resilience. Items are not disease-specific but rather measure one’s general perceived ability to recover from adversity. Participants respond using a five-point Likert scale ranging from 0 (not true at all) to 4 (true nearly all the time). Scores on the scale can range from 0 to 100, with higher scores indicating higher levels of resilience.

The Brief Resilience Scale (BRS) [15].

The BRS comprises 6 items that include both positively and negatively worded statements to gauge resilience, or the ability for patients to bounce back from stress. It utilizes a 5-point Likert scale ranging from 1 (strongly disagree) to 5 (strongly agree). Calculated scores can range from 1 to 5, with higher scores indicating higher levels of resilience.

The Inflammatory Bowel Disease Questionnaire (IBDQ) [21].

The IBDQ is a 32-item validated questionnaire to assess disease severity and quality of life in IBD, comprising four subscales: bowel health, systemic health, emotional functioning, and social functioning. Responses are given on a 7-point Likert scale, with 7 being “best function” and 1 being “worst function.” Total scores range from 32 to 224 with higher scores indicating better quality of life and lesser IBD severity.

The Brief Symptom Inventory-18 (BSI-18) [22].

The BSI-18 is an 18-item validated questionnaire that assesses overall psychological distress, symptom intensity, and total number of symptoms reported across 3 symptom dimensions: somatization, depression, and anxiety. Items on the BSI-18 are rated on a 5-point Likert scale, ranging from “not-at-all” to “extremely.” Scores range from 0 to 72 with higher scores indicating higher levels of psychological distress.

The IBD Disability Index (IBD-DI) [23].

The IBD-DI consists of 14 questions. The initial questions posed pertain to patients’ overall health, followed by questions regarding patients’ level of difficulty experienced in the past week while engaging in daily activities considering their condition. Responses are provided using a five-point Likert scale ranging from 1 (none) to 5 (very extreme) to indicate the degree of difficulty patients experience when engaging in various activities. Scores can range from 0 to 100 with higher scores indicating greater disability.

### Ethical considerations

The validation study was approved by the Institutional Review Board of the Icahn School of Medicine at Mount Sinai on August 23, 2023 (Protocol: STUDY-22-01251).

### Statistical analysis

Descriptive statistics for continuous variables are presented as mean and standard deviation (SD), while percentages and frequencies are estimated for categorical variables. Independent sample t-tests and chi-square analyses were conducted to assess significant differences in continuous and categorical data. Moreover, parametric tests were used when the data met assumptions of normality, while non-parametric tests were applied for data that did not follow a normal distribution. A statistically significant result was indicated as *P* < 0.05. Complete case analysis was conducted as no missingness was observed in our data.

### Construct validity

Construct validity, the extent to which the RISE-IBD accurately estimates the construct of interest, was assessed using factor analysis. Factor analysis is used to psychometrically validate instruments in methodological studies and is often conducted in two phases: Exploratory Factor Analysis (EFA) and Confirmatory Factor Analysis (CFA) [24]. Considering the predetermined structure of the RISE-IBD and its expected factor configuration, our initial analysis employed CFA to verify the proposed structure, comprising 17 items distributed across 3 domains.

To ensure the suitability of the data for factor analysis, the internal correlation among items in the scale were evaluated through the Kaiser-Meyer-Olkin Measure (KMO) and Bartlett’s Test of Sphericity. A KMO of at least 0.70 and a statistically significant result for Bartlett’s test were expected [25]. To assess the validity of the model, various indices with predefined criteria for evaluating goodness of fit were examined as indicated [26]. The Comparative Fit Index (CFI) and Tucker-Lewis Index (TLI) were anticipated to exceed 0.90. Root Mean Square Error of Approximation (RMSEA) ≤ 0.05 were regarded as good, while values up to 0.08 were considered fair, and > 0.08 were deemed mediocre. Lastly, Standardized Root Mean Square Residual (SRMR) < 0.08 indicated good fit.

If the hypothesized structure was found to be inadequate, the data was split in half [50–50] and EFA was conducted on the first half to explore the ideal factor structure of the resilience construct. EFA utilized a scree plot to identify the ideal number of factors above the “bend”, with factors having eigenvalues exceeding 1.00 being retained [27]. Additionally, a minimum residual method was employed to generate a model with the optimal number of factors, presenting standardized loading factors derived from a correlation matrix of the raw data. Factors with loadings ≥ 0.40 and avoiding cross-loadings > 0.30 on another factor were considered valid [28]. Following EFA, the second half of the dataset underwent CFA to evaluate the fit indices of the alternative model. The goodness-of-fit indices were examined with the criteria being the same as stated previously for CFI, TLI, and RMSEA [25].

### Item discrimination

The item-total correlation was used to evaluate whether an item effectively measured the intended resilience construct in comparison to all other items. Items with values less than 0.30 were excluded as this indicated that the item did not correlate well with the overall scale [29].

### Reliability

The internal consistency of the RISE-IBD, assessing how well the items measure different aspects of the resilience construct, was evaluated using Cronbach’s α for the entire sample (*n* = 91). A Cronbach’s alpha above 0.70 indicated adequate consistency [29]. Additionally, test-retest reliability was examined for a subset of the sample (*n* = 72) at two different time points. The test–retest reliability was assessed by an intraclass correlations coefficient (ICC). An ICC > 0.60 was considered as satisfactory and an ICC > 0.80 was deemed excellent [30]. The ICC estimate and its 95% confidence interval (CI) were calculated based on a mean rating (k = 2), 2-way mixed effects model.

### Convergent, concurrent, and divergent validity

To further assess the validity of the RISE-IBD against validated measures, Pearson’s correlation was employed for normally distributed data, while Kendall’s Tau for non-parametric and ordinal data.

Convergent validity, the extent to which resilience scores from the RISE-IBD demonstrated a strong relationship with scores on similar measures, was assessed by comparing the correlation between the RISE-IBD and two validated and widely used measures of resilience, the CD-RISC and BRS.

Concurrent validity, the degree to which resilience scores from the RISE-IBD corresponded with expected measures administered at the same point in time, was evaluated by comparing the RISE-IBD to an IBD-specific quality of life measure (i.e., IBDQ). We expected to observe positive correlations between the RISE-IBD and the CD-RISC, BRS, and IBDQ, which would justify the scale’s validity with established instruments of the same or similar constructs.

Divergent (or discriminant) validity was employed to assess whether the RISE-IBD exhibited negative correlations with measures of unrelated constructs or those theoretically distinct from resilience, yet pertinent to the experiences of individuals with IBD. We expected to observe negative correlations between the RISE-IBD and psychological distress, as assessed by the BSI-18, as well as with IBD-specific disability, as measured by the IBD-DI.

### Discriminant ability

Discriminant ability, the capacity for a measure to effectively distinguish between groups that should theoretically differ on the construct of interest, was used to investigate whether the RISE-IBD could differentiate between groups that may vary in relation to resilience. To ensure the robustness of the analysis, an exploratory data-driven approach was employed to identify relevant clinical and demographic factors.

Through preliminary analyses, variables that showed significant associations with resilience were selected for discriminant validity assessment. If the data were normally distributed, independent sample t-tests for binary variables and one-way analysis of variance (ANOVA) were used for patients across varying clinical and demographic factors. Tukey post-hoc analyses were conducted to inspect differences among groups following ANOVA. Independent sample t-test findings are reported with T-test statistic (t), degrees of freedom (df), 95% CI, and p-value. The one-way ANOVA results are reported with F-test statistic (F), df_1_, df_2_, and p-value. Tukey’s analyses comprise difference of means (D), 95% CI, and adjusted p-value (P_adj_).

### Percentile analysis

Percentile analysis is a data-driven approach that accounts for the distribution of scores within the sample, rather than relying on arbitrary or subjective criteria. To establish preliminary cutoff scores for the RISE-IBD, quartile analysis was employed to identify specific points that represented different levels of the resilience score distributions. The 25th percentile (Q1) corresponds to the value below which 25% of the observations lie, while the 50th percentile (median, Q2) represents the value below which 50% of the observations fall. Similarly, the 75th percentile (Q3) denotes the value below which 75% of the observations are found. ANOVA was employed to explore potential differences in mean RISE-IBD scores across four resilience groups, ranging from low to high resilience. Following ANOVA, Tukey’s test was utilized to discern pairwise differences between the resilience groups.

All statistical analyses were completed using RStudio (Version 2023.12.1 + 402).

## Results

### Development phase

The average BRS score among the 15 participants in the qualitative study [18] was 3.1 ± 0.50. The mean age of the participants was 45 years ± 16.2, ranging from 23 to 78 years. Many of the participants were female (67%) and non-Hispanic or Latino (87%). Regarding clinical characteristics, more than half of the participants had Crohn’s disease (60%). Eight participants had been living with IBD for more than 10 years while seven had been living with IBD for 1–5 years. Moreover, the majority reported that they were in clinical remission during the study (80%). Eight participants had undergone at least one IBD-related surgery during their lifetime.

### Validation phase

Out of the 107 participants initially interested in participating, 16 did not complete the required assessments. Therefore, our study sample comprised 91 participants with IBD who completed all the necessary validated questionnaires. Among these participants, the mean age was 39.2 ± 13.6 years (range 19–78 years), with 69.2% being women. In term of race and ethnicity, 89.0% were White, 4.4% were African American, 2.2% were Asian, and 83.5% were NOT Hispanic or Latino. Additional demographics included 46.2% were married, and 49.5% had a graduate or terminal degree. The length of time living with IBD was more than 10 years for 50.5% of participants, and 60.4% visited their IBD doctor 1–2 times per year. Furthermore, 39.6% confirmed having undergone at least one IBD-related surgery, and 69.2% reported their disease in a state of remission at the time of study participation. Demographic and clinical characteristics for the entire sample and subsamples are presented in Table 1.

**Table 1:** Demographic and clinical characteristics of participants in the validation phase

	IBD (*n* = 91)	CD (*n* = 57)	UC (*n* = 34)
	*N* (%)	*N* (%)	*N* (%)
**Age, y**			
Mean (SD)	39.2 (13.6)	40.3 (13.8)	37.2 (13.2)
Range	19.0–78.0	21.0–75.0	19.0–78.0
**Gender**			
Female	63 (69.2)	36 (63.2)	27 (79.4)
Male	28 (30.8)	21 (36.8)	7 (20.6)
**Ethnicity**			
Hispanic or Latino	13 (14.3)	6 (10.5)	7 (20.6)
NOT Hispanic or Latino	76 (83.5)	49 (86.0)	27 (79.4)
Unknown / Prefer not to say	2 (2.2)	2 (3.5)	0 (0.0)
**Race**			
Asian	2 (2.2)	1 (1.8)	1 (2.9)
Black or African American	4 (4.4)	4 (7.0)	0 (0.0)
Unknown / Prefer not to say	4 (4.4)	0 (0.0)	4 (11.8)
White	81 (89.0)	52 (91.2)	29 (85.3)
**Employment**			
Employed	61 (67.0)	36 (63.2)	25 (73.5)
Self-Employed	10 (11.0)	8 (14.0)	2 (5.9)
Student	7 (7.7)	5 (8.8)	2 (5.9)
Unemployed	13 (14.3)	8 (14.0)	5 (14.7)
**Education**			
Bachelor’s degree	27 (29.7)	14 (24.6)	13 (38.2)
Graduate or terminal degree	45 (49.5)	28 (49.1)	17 (50.0)
High school or less	6 (6.6)	6 (10.5)	0 (0.0)
Some college	13 (14.3)	9 (15.8)	4 (11.8)
**Marital status**			
Divorced	10 (11.0)	6 (10.5)	4 (11.8)
Married/domestic partnership	42 (46.2)	29 (50.9)	13 (38.2)
Single	38 (41.8)	21 (36.8)	17 (50.0)
Widowed	1 (1.1)	1 (1.8)	0 (0.0)
**Disease Duration**			
1–5 years	29 (31.9)	16 (28.1)	13 (38.2)
5–10 year	16 (17.6)	10 (17.5)	6 (17.6)
More than 10 years	46 (50.5)	31 (54.4)	15 (44.1)
**Disease Status**			
Active (in flare)	28 (30.8)	18 (31.6)	10 (29.4)
Remission	63 (69.2)	39 (68.4)	24 (70.6)
**Annual IBD Appointments**			
0 times	2 (2.2)	0 (0.0)	2 (5.9)
1–2 times	55 (60.4)	38 (66.7)	17 (50.0)
3–6 times	29 (31.9)	18 (31.6)	11 (32.4)
7 or more times	5 (5.5)	1 (1.8)	4 (11.8)
**Experienced IBD-related Surgery**			
Yes	36 (39.6)	29 (50.9)	7 (20.6)
No	55 (60.4)	28 (49.1)	27 (79.4)
**Experienced Bowel Resection**			
Yes	23 (25.3)	22 (38.6)	1 (2.9)
No	68 (74.7)	35 (61.4)	33 (97.1)

### Psychometric properties

#### Construct validity

**Fig. 1:**
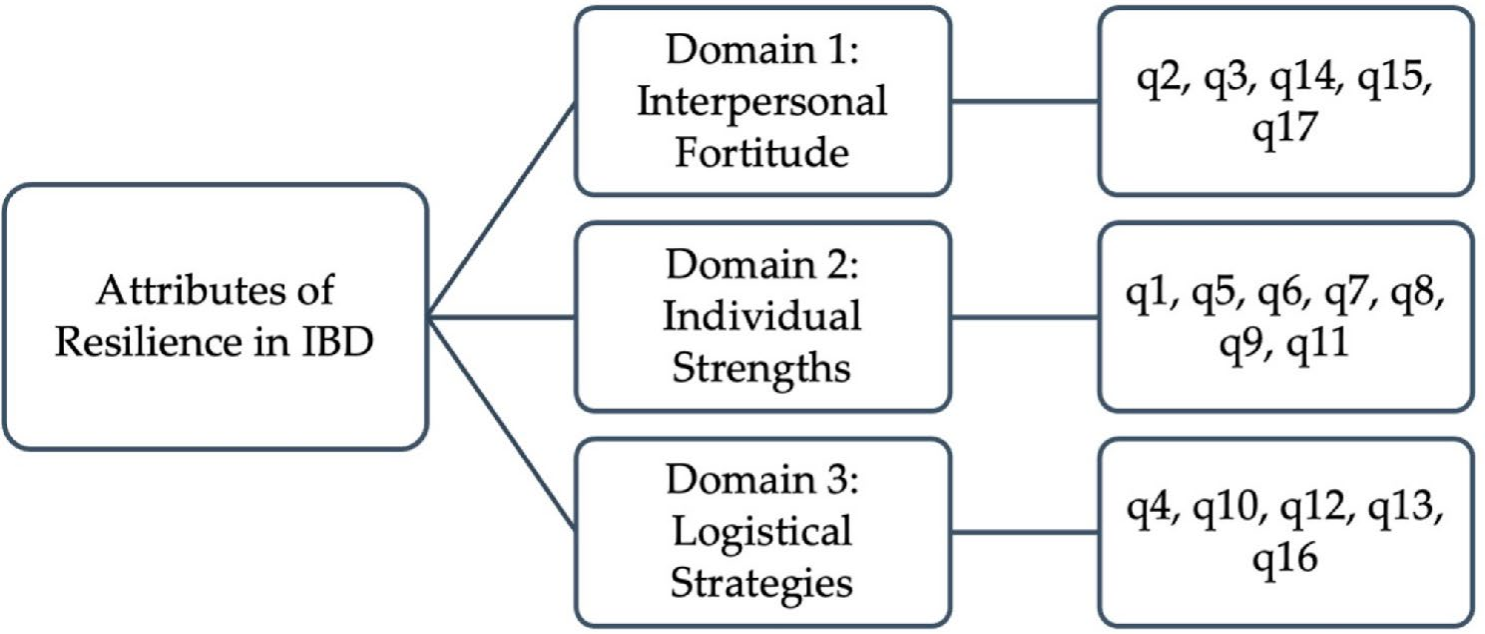
Hypothesized RISE-IBD Model: A 17-item, 3-domain framework representing key attributes of resilience in individuals with IBD

The initial 17-item RISE-IBD exhibited adequate sampling with a KMO index of 0.79 and Bartlett’s test of sphericity was significant (χ2 (136) = 486.23, *P* < 0.001). This indicated sufficient evidence to proceed with factor analysis. The CFA performed on the hypothesized model (Fig. 1) yielded a CFI of 0.76 and TLI of 0.72. Additionally, the RMSEA and SRMR were 0.092 and 0.095, respectively.

To enhance model fit, modification indices were computed, and those with the highest modification index (MI) among variables within the same latent group were included in the hypothesized model. Incorporating 5 indices into the original model yielded the following results: CFI = 0.82, TLI = 0.77, RMSEA = 0.082, and SRMR = 0.093. These findings did not significantly enhance the model and further indicated inadequate fit of the hypothesized framework, emphasizing the necessity to explore alternative models. Upon performing item discrimination of the 17-item RISE-IBD, it was observed that item 16 had the lowest item correlation at 0.24. Additionally, items 15 and 17 also contributed minimally to the resilience construct and were thus removed from the model. This resulted in a refined 14-item RISE-IBD that was further psychometrically tested.

**Fig. 2:**
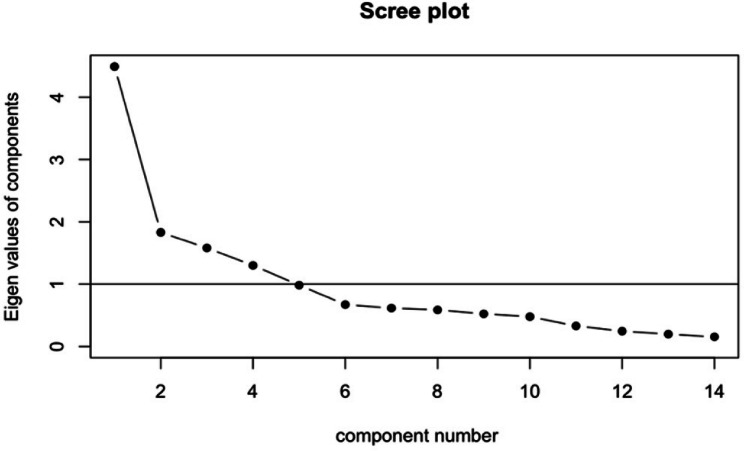
A scree plot generated from the EFA provides a visual depiction of the eigenvalues of the components (latent factors) extracted during the analysis. Four eigenvalues exceed the 1.00 threshold line indicating the optimal number of factors to retain for additional factor evaluation

**Fig. 3:**
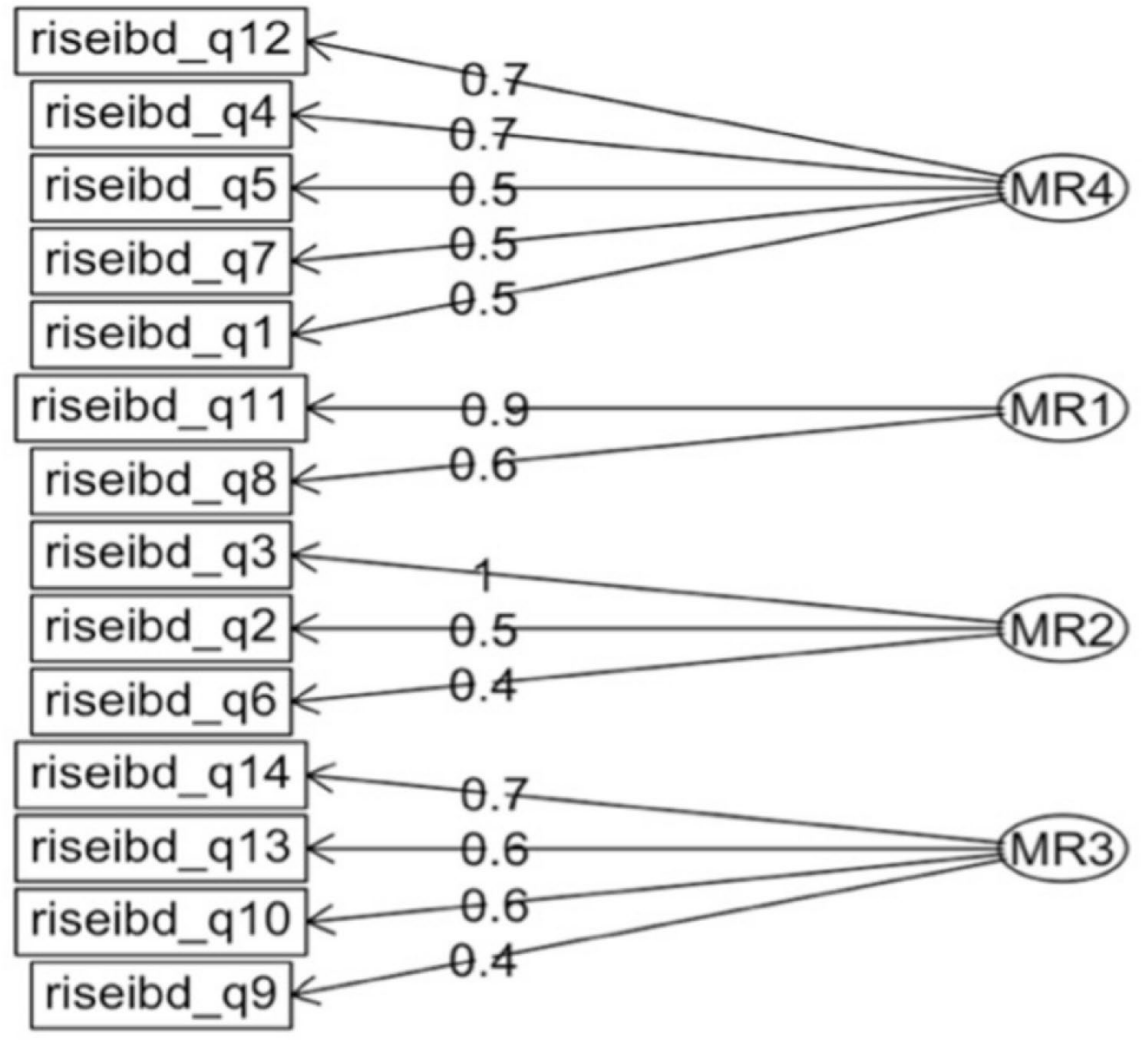
The EFA model presents a dynamic 4-factor representation for the measurement construct of resilience. The values provided to the right of each of the 14 items indicate item-factor loadings which represent standardized regression coefficients. These loadings, which can range from − 1 to + 1, indicate the strength and direction of the relationship between each item and its corresponding factor

The 14-item RISE-IBD exhibited a KMO index of 0.79 and Bartlett’s test yielded statistically significant results (χ2 (91) = 288.38, *P* < 0.001). EFA was used on the first split-half of the data to uncover the optimal underlying structure for the construct of resilience. A generated scree plot (Fig. 2) indicated the presence of four factors with eigenvalues exceeding 1.00. A fifth factor had an eigen value slightly below the 1.00 threshold and therefore was not considered. Using a minimum residual method, a random four-factor model was developed (Fig. 3) that demonstrated a TLI of 1.01 and RMSEA index of 0. Additionally, the proposed model contained all factors loading to at least 0.40 with none overlapping further affirming a satisfactory fit of the data. These findings supported the decision to proceed with CFA on the suggested structure. Using the remaining half of the data, CFA was employed that provided improved goodness of fit indices: CFI = 0.94, TL1 = 0.92, and RMSEA = 0.054. These findings suggested that the model fits significantly better than the initially hypothesized structure.

Upon examining the correlations among the 4 latent variables (or domains), all domain correlations exceeded 0.60. This suggests that the 4 domains are interconnected with changes in one domain moderately to strongly linked with changes in the others. As a result, the 14-item RISE-IBD was identified as the optimal model structure for evaluating resilience in IBD and underwent further evaluation.

#### Internal reliability

Based on the entire sample, the 14-item RISE-IBD exhibited excellent internal consistency with a Cronbach’s α of 0.82. Good internal reliability was also confirmed for the 4 domains of the RISE-IBD with a range of Cronbach’s α from 0.50 to 0.71. Table 2 presents the means and standard deviations of all items, along with item–total correlations ranging from 0.36 to 0.70 (*P* < 0.001).

**Table 2:** The final 14-item RISE-IBD descriptions, item statistics, and item-Total score correlations

Item Description	Mean (SD)	Correlations*
1. Staying determined despite challenges	3.2 (0.69)	0.57
2. Being confident in one’s support system	3.3 (0.86)	0.56
3. Not feeling a sense of belonging	2.4 (1.03)	0.59
4. Proactive about one’s medical management	3.2 (0.79)	0.36
5. Celebrating successes	2.6 (1.14)	0.44
6. Difficulty staying calm	2.2 (1.03)	0.62
7. Feeling a sense of purpose	3.1 (0.88)	0.62
8. Struggling to accept one’s condition	2.5 (1.21)	0.49
9. Being kind to oneself	2.6 (0.82)	0.70
10. Being able to move on from challenges	3.0 (0.80)	0.64
11. Believing in living a good life	3.1 (0.89)	0.64
12. Using helpful strategies when stressed	2.5 (0.97)	0.48
13. Comfortable adjusting plans	2.6 (0.93)	0.56
14. Difficulty setting boundaries with people.	2.2 (1.15)	0.52

*All item-total score correlations were significant at *P* < 0.001

#### Test-retest reliability

Within a subset of the sample (*N* = 72), participants completed the measure at two distinct timepoints, with an average of 49.9 days between administrations (range: 25–132 days). The ICC from timepoint 1 to 2 was excellent at 0.91 [95% CI (0.86, 0.94), *P* < 0.001].

#### Convergent, concurrent, and divergent validity

The validity of the 14-item RISE-IBD scale was further evaluated through its relationship with other pertinent questionnaires. To establish convergent validity, the RISE-IBD exhibited positive correlations with total scores on the CD-RISC (*r* = 0.74, *P* < 0.001) and BRS (*r* = 0.59, *P* < 0.001). Regarding concurrent validity, the RISE-IBD displayed a positive correlation with the IBDQ (τ = 0.33, *P* < 0.001). Divergent validity was observed as the RISE-IBD demonstrated negative correlations with the BSI-18 (τ = -0.36, *P* < 0.001) and IBD-DI (τ = -0.30, *P* < 0.001).

#### Discriminant ability

To evaluate the discriminant ability of the RISE-IBD, exploratory analyses was conducted to identify significant clinical and demographic variables and subsequently examine whether the measure could distinguish between groups differing on these factors. The ANOVA revealed a marginally significant effect of employment status [F(3, 87) = 2.6, *P* = 0.055] on RISE-IBD scores. For employment status, Tukey’s post-hoc comparisons also revealed significant differences between Unemployed and Employed groups [D = -5.7, 95% CI (-11, -0.22), P_adj_ = 0.038]. As the data for Race were non-normally distributed, a Kruskal-Wallis test was used to assess group differences in RISE-IBD scores (χ²(3) = 8.92, *p* = 0.030). Post hoc analysis using Dunn’s test with Bonferroni correction revealed a significant difference between Black or African American and White participants (Z = -2.76, P_adj_ = 0.035).

The results of the independent sample t-test indicated significantly lower mean RISE-IBD scores across individuals exhibiting chronic pain [t(89) = -2.1, 95% CI (-6.5, -0.22), *P* = 0.036] and anxiety [t(89) = -3.4, 95% CI (-7.5, -1.9), *P* = 0.001] compared to those not exhibiting these symptoms. There were also significant differences in higher mean resilience scores between individuals using anti-TNF medications such as adalimumab (Humira), golimumab (Simponi), certolizumab pegol (Cimzia), and infliximab (Remicade), [t(89) = 2.0, 95% CI (0.0013, 5.9), *P* = 0.050]. Users of immunomodulators including mercaptopurine (Purinethol), methotrexate (Trexall), and azathioprine (Imuran) had lower mean RISE-IBD scores, [t(89) = -2.5, 95% CI (-14, -1.5), *P* = 0.015].

In recognizing that the study’s findings are preliminary, additional exploratory analysis with an alpha of 0.10 was set to identify any additional trends that may warrant further investigation involving IBD-related resilience. A significant relationship was observed between lower mean RISE-IBD scores and key self-reported comorbidities and medication use, including the presence of fatigue [t(89) = -1.7, 95% CI (-5.4, 0.40), *P* = 0.090], depression [t(89) = -1.8, 95% CI (-7.3, 0.28), *P* = 0.069], and use of anti-anxiety medication [t(89) = -1.9, 95% CI (-6.5, 0.23), *P* = 0.068].

#### Percentile analysis

**Fig. 4:**
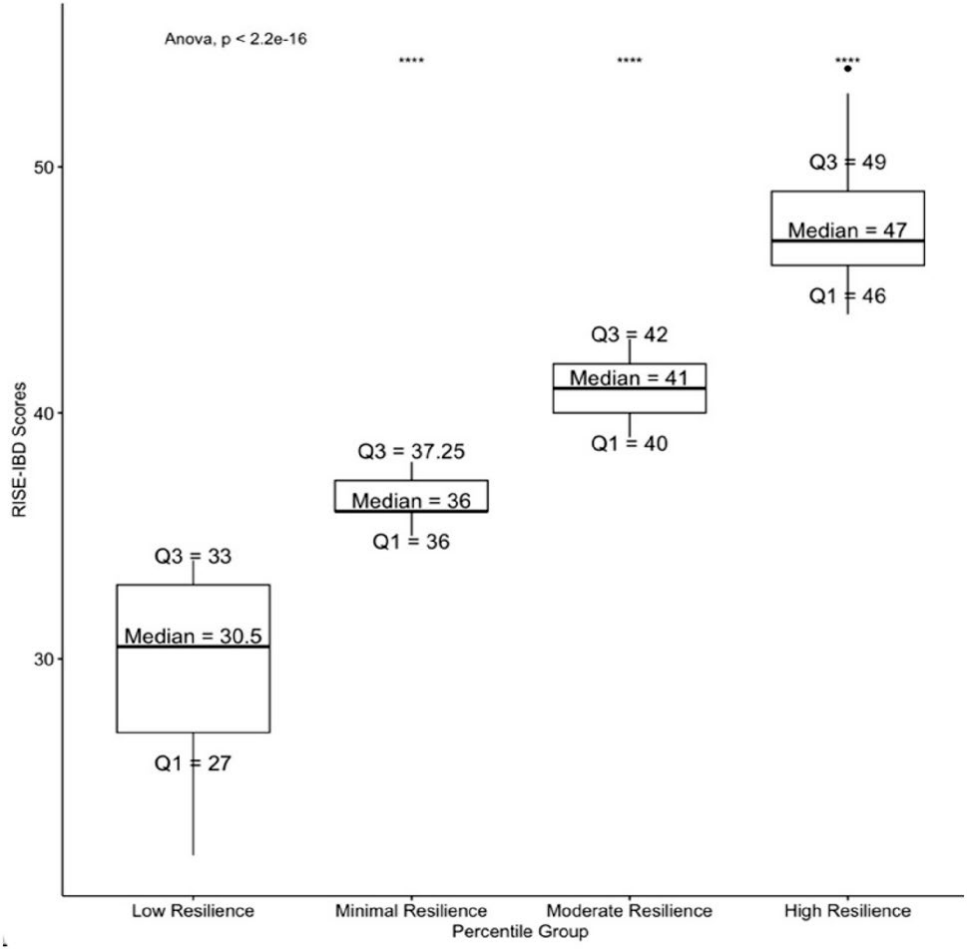
Four resilience groups were defined using percentile analysis of the RISE-IBD scores distribution. The vertical box plots illustrate the median (center horizontal line), interquartile range (25th percentile [Q1] and 75th percentile [Q3]), and range of scores for each group. Whiskers (vertical lines) extend from the box’s outer edges to the most extreme point within 1.5 times the interquartile range (Q3-Q1). Any observations beyond this range are depicted as individual points. All Tukey pairwise comparisons with “Low Resilience” as the reference group are statistically significant (**** = *P* < 0.001)

Quartile values were computed as follows: Q1 = 34, Q2 = 38, and Q3 = 43. Based on this data, we categorized resilience scores into the following groups: scores ranging from 0 to 34 were classified as ‘Low Resilience’, scores from 35 to 38 as ‘Minimal Resilience’, scores from 39 to 43 as ‘Moderate Resilience’, and scores from 44 to 54 as ‘High Resilience’. Tukey’s pairwise comparisons revealed that all differences in mean RISE-IBD scores, with ‘Low Resilience’ serving as the reference group, were statistically significant (P_adj_ < 0.001). These comparisons are illustrated in Fig. 4.

## Discussion

### Interpretation of findings

Previous research has demonstrated the promising role of resilience, typically assessed using generic measures like the CD-RISC, in improving physical health and overall well-being for individuals with IBD [7, 9]. Nevertheless, a standardized definition of resilience tailored to the IBD patient demographic has yet to be established. Psychological resilience, in the context of IBD, is certainly a multifaceted construct shaped by both internal (personal) and external (environmental) factors [18, 31]. Accordingly, the goal of this study was to provide preliminary evidence for the validation of a patient-centered resilience tool that could evaluate IBD-related *resilience* while taking such factors into account.

**Fig. 5:**
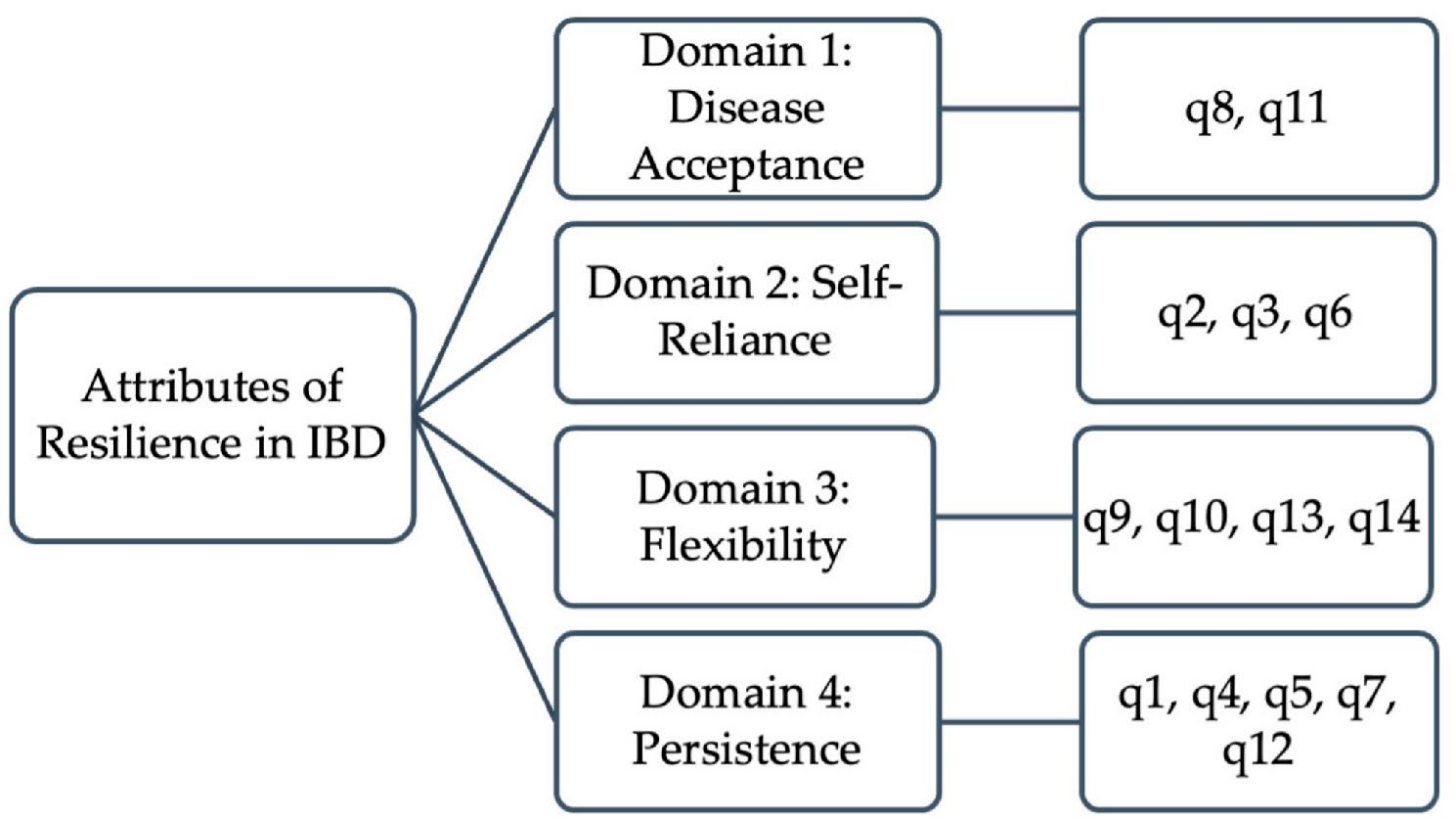
Final RISE-IBD Model: A 14-item, 4-domain framework representing key attributes of resilience in individuals with IBD

A qualitative research study was employed to explore attributes of resilience as defined by 15 patients with IBD. Factor analysis was used to evaluate the internal construct validity of the 17-item RISE-IBD, where the sample-to-item ratio was expected to be at least 5:1 [32]. With this criterion, the minimum sample size required for the validation study was 85 participants. In our 91-sample study, CFA was initially used to evaluate the validity of the hypothesized archetype. Per the literature, when a CFA model does not fit well, including significantly large MI into the structure allows chi-square statistics to be reduced, thus yielding a better statistically fitted result [33]. Despite the inclusion of 5 MI, the model continued to exhibit poor fit, prompting the utilization of item discrimination to scrutinize and reduce the scale’s items to 14. With no predetermined assumptions regarding the number of factors, or the loading of items onto which factors, EFA was conducted on the 14-item RISE-IBD. The generated scree plot guided our research team in devising a 4-factor model. The contents of each factor were assigned appropriate names based on their constituent items (Fig. 5).

The results from the discriminant ability indicated variations in mean resilience scores, measured using the RISE-IBD, across demographic and clinical factors. In terms of race, resilience scores averaged higher for individuals identifying as White compared to those from Black or African American demographic groups. Furthermore, resilience scores were lower for individuals in the Unemployed group compared to those in the Employed group. Such findings reflect a potential disparity in access to resources, socioeconomic factors, or cultural influences that may impact resilience levels. Indeed, individuals from racial and ethnic minority groups, or those with lesser financial stability, may have difficulty seeking and obtaining mental health care [34]. Therefore, it is important to not only develop community outreach programs to support resilience-building efforts for minority groups, but also to tailor such programs to individuals with IBD given their unique racial, ethnic, and financial background.

As anticipated, participants with chronic pain and anxiety exhibited lower RISE-IBD scores compared to those without these comorbidities. Remarkably, individuals using anti-TNF medications exhibited higher resilience scores, whereas those using immunomodulators had lower resilience scores. These findings confirm previous literature suggesting that resilience might mediate the connection between perceived autonomy support and medication adherence [35]. However, it is also possible that higher resilience in anti-TNF users might reflect disease control (e.g., a state of remission) rather than treatment effect. Therefore, healthcare professionals should collaborate with patients to assess their overall well-being comprehensively, considering not only IBD-related medications but also psychological interventions alongside them to control disease status and augment resilience. While exploratory findings did not attain the conventional level of significance, adopting an alpha level of 0.10 enabled us to explore potentially significant trends. Aligning with former research findings, individuals with IBD who were dealing with depression and those using anti-anxiety medications exhibited lower resilience scores in comparison to those without these comorbidities [36]. Overall, the results from the discriminant ability suggest that the RISE-IBD may be helpful in assessing differences in demographic and clinical factors, highlighting its potential utility in assessing various aspects related to IBD.

### Practical applications

The 14-item RISE-IBD assesses resilience across four distinct domains. ***Disease Acceptance*** measures individuals’ acknowledgment and acceptance of the presence of IBD in their lives. ***Self-Reliance*** measures the degree of empowerment individuals feel in managing their disease independently and seeking personalized strategies, such as mindfulness practices, to help manage their condition. ***Flexibility*** evaluates the ability for patients to adapt to the uncertainties of living with IBD, as well as establishing boundaries with people, particularly during disease flares, treatment adjustments, and lifestyle changes. ***Persistence*** evaluates patients’ capacity to maintain optimism when pursuing goals despite setbacks posed by their IBD. Moreover, it emphasizes patients’ ability to persist in medically managing their condition and to seek aid from trusted individuals or providers when facing uncertainty related to their IBD.

By measuring resilience across such domains, healthcare providers can comprehensively assess resilience and tailor interventions appropriately. For instance, patients scoring low in the Disease Acceptance domain may require targeted counseling to address any misconceptions about their IBD and should gradually work towards embracing their condition as an integral part of their identity. Patients demonstrating high levels of Self-Reliance are likely to adhere to treatment plans and engage in self-care or stress-reduction techniques, allowing them to carefully regulate their emotions in the face of adversity. To enhance Flexibility, healthcare providers can educate patients on the comorbidities often associated with their IBD, therefore enabling patients to adapt to changes with their health and make informed decisions. Providing guidance on effective communication is also crucial to helping patients establish boundaries in social or work settings when needed. For individuals with low Persistence, providers can assist their patients in creating S.M.A.R.T. (Specific, Measurable, Achievable, Relevant, and Time-sensitive) goals. By implementing this technique, for personal or medical goals, patients with IBD can feel empowered to pinpoint specific tasks they should accomplish judiciously and within a specific time-frame, thus facilitating more focused efforts and increasing the probability of successful outcomes [37].

The utilization of the RISE-IBD could also be used to identify patients who may be at higher risk for poor outcomes such as decreased quality of life, enhanced anxiety, increased disease activity, and more susceptibility to IBD-related surgeries (particularly for those with CD), due to lower levels of resilience [9]. Such patients would require more frequent follow-up visits or early intervention to prevent disease exacerbations. Likewise, the measure can be used to longitudinally monitor changes in resilience in response to medical or psychological interventions, and even life events. This information would help healthcare providers measure the effectiveness of treatments and adjust them as needed. For instance, improvements in resilience may indicate that a patient is responding well to treatment and experiencing better quality of life, whereas declines in resilience may signal the need for additional support or adjustments to therapy.

By incorporating resilience assessments into routine clinical practice, healthcare providers can engage patients in shared decision making and empower them to play an active role in their care. Patients could also use their resilience scores as a tool to track their progress, set goals, and make informed decisions about their treatment options, following a similar framework to that of the GRITT™ method [38]. This collaborative approach can lead to better treatment adherence, improved patient satisfaction, and ultimately, better health outcomes for individuals living with IBD.

### Strengths

The overall reliability of the 14-item RISE-IBD was excellent with a Cronbach’s alpha of 0.82. Particularly, items 2,7, and 11 had mean scores above 3.0 indicating that participants felt more strongly with having a sense of confidence in their support systems, feeling a sense of purpose living with IBD, and believing they could live a fulfilling life despite the challenges their IBD posed (see Table 2). These sentiments align with the positive psychology concept of optimism or “meaning finding,” traits associated with reduced stress and depression, as well as a higher likelihood of experiencing positive healthcare outcomes [8].

To further assess the consistency of the construct without any added intervention, participants were administered a re-test of the RISE-IBD. In contrast to the typical duration of 4 to 6 weeks observed in most test-retest reliability studies, our participants completed the re-test on average within 7 weeks. This extended interval allowed for a more robust assessment of the measure’s replicability, providing insights into its long-term stability. Moreover, with an ICC of 0.91 and 95% CI (0.86, 0.94), the findings suggest an excellent and significant level of internal consistency in the responses collected, supporting the scale’s potential for assessing resilience at two distinct time points. By demonstrating convergent, concurrent, and divergent validity, we confirmed existing relationships between resilience and associated constructs in IBD. With a strong correlation to the CD-RISC, a sound psychometric tool capable of discerning between those with greater and lesser resilience, the RISE-IBD similarly demonstrates its ability to evaluate psychological attributes associated with resilience, including coping strategies and overall well-being [14]. Moreover, while the CD-RISC was limited in its ability to acknowledge the nuances of resilience across different domains, such as the varied impacts of interpersonal and personal relationships on the resilience construct, [14] the RISE-IBD addresses these factors explicitly.

A statistically significant and positive correlation emerged between the RISE-IBD and the IBDQ, not only affirming concurrent validity, but also confirming previous study findings that identified positive relationships between resilience and QOL in the IBD population [7, 9]. Certainly, those with greater resilience may be more likely to self-manage their condition and utilize specific coping mechanisms to mitigate the impact of IBD-related stressors, therefore enhancing their QOL. Furthermore, the results of divergent validity revealed a significant and negative correlation between resilience and psychological distress, as assessed by the BSI-18. This supports previous research suggesting that resilience may serve as a protective factor against anxiety and depression. It also highlights the potential benefits of using resilience-based coping strategies, rooted in positive psychology, to enhance psychosocial well-being among individuals with IBD [8, 9].

Lastly, a statistically significant and inverse correlation was found between the RISE-IBD and IBD-specific disability, as assessed by the IBD-DI. Such finding underscores the potential applicability of resilience for individuals with disabilities in serving as a resource to help them cope positively to their disabilities when confronted with various lifestyle changes [39]. By fostering resilience, individuals with disabilities may be able to better adapt to new circumstances and navigate the challenges associated with their conditions more effectively.

### Limitations

In the qualitative study, participants were selected based on moderate resilience scores to ensure that they could clearly articulate their coping strategies and provide detailed, constructive definitions of resilience in IBD. As noted in a previous study, this sampling strategy may have introduced selection bias and limited the generalizability of individuals and their resilience scores [18]. Likewise, given that half of the validation cohort had been living with IBD for more than 10 years, the psychometric properties of the RISE-IBD may differ in newly diagnosed patients. Future studies should assess the scale’s performance in newly diagnosed patients, as resilience may manifest differently earlier in the disease course. This could shed light on whether resilience in IBD reflects a learned or adaptive process, or if it represents an innate, trait-like characteristic within this population. Moreover, correlations with depression may be stronger among individuals who are newly diagnosed, as they may not have yet developed effective coping mechanisms to manage the emotional impact of their IBD. Therefore, resilience-building interventions would play a critical role in mitigating depressive symptoms particularly during this time.

The quantitative study also encountered several limitations. Firstly, the sample size was relatively small compared to most validation studies that follow a sample-to-item ratio of 10:1 [40]. Given the limited sample size, splitting of the data for EFA and CFA did not yield splits that were perfectly consistent with one another and therefore may not have captured the diversity of the entire dataset. As such, future studies that replicate the splitting of the data should use a sufficiently larger sample size to ensure that each split maintains adequate variance. Moreover, the study’s sample was predominantly comprised of White and Non-Hispanic females with CD, and about half of the participants had graduate or terminal degrees, affecting the generalizability of the findings. Patients seeing community gastroenterologists or those without access to an IBD-specific clinic may have limited access to specialized resources for their conditions. Future studies should include participants from more demographically diverse backgrounds and be conducted across both regional and tertiary care centers.

Secondly, the cross-sectional design used in the validation study restricted data collection to a single point in time and prevented the assessment of temporal changes in resilience. As such, the design of the study prohibited the ability to establish causal relationships with respect to resilience. Longitudinal studies using the RISE-IBD would allow researchers to observe potential fluctuations in resilience scores and assess the association of resilience with added behavioral or psychosocial interventions to improve patient outcomes.

Another limitation was that disease activity status was based solely on patient self-reported data. As objective clinical or biochemical markers (e.g., fecal calprotectin, CRP) were not available, future studies would benefit from incorporating clinical assessments to evaluate how resilience may be influenced by, or related to, disease activity across key resilience domains. Lastly, it is crucial to acknowledge the exploratory nature of the factor analysis that was performed, indicating that the 14-item scale may require further psychometric testing and validation by other researchers and practitioners.

## Conclusion

In summary, the RISE-IBD, consisting of 14 items across 4 domains, may be a potentially valid and reliable instrument for measuring resilience among individuals with IBD. Employing an assessment of each domain would enable practitioners to identify patients’ areas of vulnerability and suggest tailored interventions aimed at enhancing resilience to ultimately improve IBD-related outcomes. Subsequent research should focus on enhancing the psychometric robustness of the scale by administering it to different patient samples and conducting additional factor analyses to contribute to its validation. By continually refining and validating the RISE-IBD, we can reinforce its utility as a pivotal tool in the ongoing effort to optimize the resilience of individuals navigating the challenges of IBD, therefore leading to improved QOL.

## Data Availability

The datasets used and/or analyzed during the current study are available from the corresponding author on reasonable request.

## Abbreviations

IBD: Inflammatory bowel disease
CD: Crohn’s disease
UC: Ulcerative colitis
US: United States
HRQOL: Health-related quality of life
QOL: Quality of life
CD-RISC: Connor–Davidson Resilience Scale
BRS: Brief Resilience Scale
RISE-IBD: Resilience Scale for Inflammatory Bowel Disease
FGDs: Focus Group Discussions
IBDQ: Inflammatory Bowel Disease Questionnaire
BSI-18: Brief Symptom Inventory-18
IBD-DI: IBD Disability Index
SD: Standard deviation
EFA: Exploratory Factor Analysis
CFA: Confirmatory Factor Analysis
KMO: Kaiser-Meyer-Olkin Measure
CFI: Comparative Fit Index
TLI: Tucker-Lewis Index
RMSEA: Root Mean Square Error of Approximation
SRMR: Standardized Root Mean Square Residual
ICC: Intraclass correlation coefficient
CI: Confidence interval
ANOVA: Analysis of variance
t: T-test statistic
df: Degrees of freedom
F: F-test statistic
P_adj_: Adjusted p-value
MI: Modification index
